# Challenges and Opportunities of Human Immunodeficiency Virus, Hepatitis B Virus and Hepatitis C Virus Control and Elimination Policies, Regulations, and Strategies in Ethiopia

**DOI:** 10.1002/hsr2.73435

**Published:** 2026-10-26

**Authors:** Mequanente Dagnaw, Bisrat Misganaw Geremew, Lemma Derseh Gezie

**Affiliations:** ^1^ Department of Epidemiology and Biostatistics, Institute of Public Health University of Gondar Gondar Ethiopia; ^2^ Department of Medical Biotechnology, Institute of Biotechnology University of Gondar Gondar Ethiopia

**Keywords:** elimination, Ethiopia, hepatitis B, hepatitis C, HIV, policy, regulation, stakeholders, strategy, viral hepatitis

## Abstract

**Background:**

Human immunodeficiency virus (HIV), hepatitis B virus (HBV), and hepatitis C virus (HCV) remain major public health challenges globally and in Ethiopia. Although policies, regulations, and strategies have been developed to support their control and elimination, translating these policy commitments into effective implementation remains challenging. Understanding stakeholder experiences and perspectives is important for identifying gaps in policy awareness, participation, implementation, and program coordination.

**Objective:**

This study aimed to explore the challenges and opportunities affecting the development, implementation, and effectiveness of HIV, HBV, and HCV control and elimination policies, regulations, and strategies in Ethiopia.

**Methods:**

A facility‐ and institution‐based qualitative study was conducted among stakeholders involved in HIV, HBV, and HCV policy development, implementation, regulation, monitoring, research, and service delivery. Thirty‐four participants were purposively selected based on their professional roles, experience, knowledge, and involvement in relevant programs. Data were collected from April 17 to May 27, 2023, using qualitative interviews and focus group discussions, complemented by a review of relevant policy, strategy, guideline, and program documents and observations of selected service‐delivery and pharmaceutical settings. Data were transcribed, translated where necessary, and thematically analysed using MAXQDA Analytics Pro 2024. The analysis focused on policy awareness, stakeholder involvement, stakeholder roles, implementation status, opportunities, and challenges.

**Results:**

Participants had a mean work experience of 16.82 years (SD = 0.79), and 30 (88.2%) were male. Of the 34 participants, 12 (35.3%) clearly identified or described the existence and key features of HIV, HBV, and HCV control and elimination policies, regulations, and strategies, while 22 (64.7%) did not. Fifteen (44.1%) participants reported stakeholder involvement in policy and strategy development, whereas 19 (55.9%) did not report adequate opportunities for participation. Most participants, 32 (94.1%), demonstrated an understanding of stakeholder roles and responsibilities in policy development and implementation. However, only 8 (23.5%) participants reported a clear current implementation status or progress, while 26 (76.5%) reported no clear implementation status. Twenty‐nine (85.3%) participants reported limited or unclear opportunities for strengthening HIV, HBV, and HCV control and elimination. Nearly all participants, 32 (94.1%), identified challenges affecting policy and strategy implementation, including COVID‐19‐related disruptions and concerns regarding the translation of policies and strategies into practice.

**Conclusion:**

The findings indicate a substantial gap between recognition of stakeholder responsibilities and the practical awareness and implementation of HIV, HBV, and HCV control and elimination policies, regulations, and strategies in Ethiopia. Strengthening policy dissemination, inclusive stakeholder engagement, implementation monitoring, coordination, and the translation of policy commitments into service‐delivery activities may support progress toward national and global elimination targets. Sustainable financing, strengthened health‐system capacity, stakeholder accountability, and reliable monitoring and evaluation systems are important considerations for improving implementation.

**Abbreviations**
APHI: Amhara Public Health Institute
ARV: antiretroviral
CSOs: Civil Society Organizations
EBV: Epstein‐Barr virus
EGA: Ethiopian Gastroenterology Association
EPHA: Ethiopian Public Health Association
EPHI: Ethiopian Public Health Institute
FMOH: Federal Ministry of Health
GHSS: Global Health Sector Strategy
HBV: hepatitis B virus
HCV: hepatitis C virus
ICAP: International Centre for Aids Care and Treatment Program
IPC: infection, prevention & control
MoH: Ministry of Health
M&E: monitoring & evaluation
NGO: None Government organizations
NTWG: National Technical Working Group
NTWG: National Technical Working Group
SBCC: social behavioral change communication
SD: standard deviations
SDG: sustainable development goals
SPSS: statistical package for social science
SWOT: strength weakness opportunity & threat
TB: tuberculosis
TWG: technical working group
WASH: water, sanitation and hygiene
WHA: World Hepatitis Alliance
WHO: World Health Organizations

## 1 Introduction

Human immunodeficiency virus (HIV), hepatitis B virus (HBV), and hepatitis C virus (HCV) remain major public health challenges worldwide and continue to cause substantial morbidity, mortality, and health‐system burden. HBV and HCV can cause acute and chronic liver disease, cirrhosis, and hepatocellular carcinoma, while HIV requires lifelong treatment and sustained healthcare services. These infections also share important prevention and health‐system priorities, including safe blood and injection practices, prevention of mother‐to‐child transmission, testing, treatment, monitoring, community engagement, and access to quality health services. Despite major advances in prevention, diagnosis, and treatment, gaps remain in translating available interventions into effective population‐level program [1, 2]. In 2022, approximately 254 million people were living with chronic HBV infection and 50 million with HCV infection globally, and viral hepatitis B and C caused approximately 1.3 million deaths, mainly from cirrhosis and liver cancer [2].

The burden of viral hepatitis is particularly important in low‐ and middle‐income countries and in the WHO African Region. Although effective prevention, diagnostic, and treatment interventions are available, access remains inadequate in many settings. WHO's Global Health Sector Strategies 2022–2030 established the goal of eliminating viral hepatitis B and C as public health threats by 2030 and emphasized people‐centered services, health‐system strengthening, strategic information, partnerships, community engagement, and integrated approaches to service delivery [1]. However, recent evidence demonstrates that the global response remains off track. The 2026 WHO Global Hepatitis Report estimated that approximately 1.3 million people died from HBV‐ and HCV‐related cirrhosis and liver cancer in 2024, while treatment coverage for chronic HBV remained below 5% despite the availability of effective antiviral therapy [3]. These findings indicate that the availability of effective interventions alone is insufficient without appropriate policies, financing, governance, stakeholder coordination, monitoring, and implementation systems.

Ethiopia has a substantial burden of HBV and HCV and has progressively developed policy and strategic responses to address viral hepatitis. Earlier systematic evidence estimated the pooled prevalence of HBV infection in Ethiopia at 7.4% [3], while an updated systematic review reported a pooled prevalence of approximately 6% [4]. More recent evidence continues to demonstrate a considerable HBV burden, with a systematic review of studies conducted in Ethiopia reporting a pooled prevalence of 6.9%. Previous Ethiopian studies have also identified important gaps in prevention, diagnosis, treatment, surveillance, and health‐system capacity.

Ethiopia has recognized viral hepatitis as an important public health problem and has developed policies, strategies, and program structures to address it. A landmark qualitative assessment examined the national response to HBV and HCV using interviews with key informants, policy and strategy document review, and observation of pharmaceutical premises [5]. The study found that Ethiopia was developing strategic plans and guidelines but faced limited partnerships and resource mobilization, inadequate community awareness, restricted availability of screening, insufficient treatment guidance, weak surveillance, and limited availability of effective medicines. The authors concluded that the national response was fragmented and recommended strengthening surveillance, screening, vaccination, treatment, policy guidance, partnerships, and access to affordable medicines. Subsequent research has identified additional opportunities for HCV elimination in Ethiopia, including phased micro‐elimination, task shifting, improved access to affordable diagnostics and direct‐acting antivirals, blood‐safety interventions, and multisectoral collaboration [6].

Despite these developments, existing HIV, HBV, and HCV policies, regulations, and strategies are understood and experienced by stakeholders responsible for their development and implementation at different levels of the Ethiopian health system. In particular, limited recent evidence is available regarding stakeholders’ awareness of existing policies and strategies, their participation in policy development, understanding of roles and responsibilities, perceptions of current implementation status, available opportunities, and challenges affecting implementation. The 2016 Ethiopian policy assessment provided important baseline evidence regarding the national hepatitis response, but the policy and program environment has subsequently evolved, including the development of newer strategic frameworks. Therefore, there is a need for updated evidence examining whether policy commitments are visible and understood by stakeholders and how they are translated into implementation across national, regional, district, and service‐delivery levels.

There is limited stakeholder‐level evidence linking the formal existence of HIV, HBV, and HCV policies, regulations, and strategies with their practical implementation, coordination, monitoring, and perceived effectiveness in Ethiopia. The existence of policy documents does not necessarily demonstrate that stakeholders are aware of them, participate in their development, understand their responsibilities, or have adequate resources and institutional mechanisms to implement them. This gap is particularly important because WHO identifies strategic information, accountability, partnerships, financing, community engagement, and integrated service delivery as essential components of achieving the 2030 HIV and viral hepatitis targets [1]. The continuing global shortfall in progress toward viral hepatitis elimination further demonstrates the importance of understanding implementation barriers at country level.

To address this gap, the present study explored the challenges and opportunities of HIV, HBV, and HCV control and elimination policies, regulations, and strategies in Ethiopia. The study examined perspectives from stakeholders involved in policy development, program management, research, regulation, laboratory and pharmaceutical services, healthcare delivery, and other relevant activities. Qualitative interviews and focus group discussions were complemented by review of relevant policies, strategies, guidelines, and program documents and observations of selected health‐service and pharmaceutical settings. Specifically, the study assessed stakeholders’ awareness of policy and strategy existence and features, involvement in policy development, understanding of roles and responsibilities, perceptions of current implementation status, available opportunities, and challenges affecting implementation. By addressing these dimensions, the study provides updated evidence to inform policymakers, program managers, health planners, researchers, professional associations, healthcare providers, and development partners in strengthening policy dissemination, stakeholder engagement, coordination, implementation, monitoring, and accountability toward HIV, HBV, and HCV control and elimination in Ethiopia.

## 2 Methods

### 2.1 Study Setting and Period

The study was conducted at selected national, regional, and subnational institutions in Ethiopia that are directly involved in the development, implementation, regulation, monitoring, and delivery of Human Immunodeficiency Virus (HIV), Hepatitis B Virus (HBV), and Hepatitis C Virus (HCV) control and elimination services. The study sites included the Ministry of Health (MoH), Ethiopian Public Health Institute (EPHI), Amhara Public Health Institute (APHI), regional health bureaus, zonal health offices, district health offices, University of Gondar Comprehensive Specialized Hospital, selected health centers, drug stores, and regional blood bank units.

These institutions were selected because they represent key stakeholders involved in policy formulation, program coordination, disease surveillance, prevention, diagnosis, treatment, laboratory services, pharmaceutical supply, and implementation of HIV, HBV, and HCV control and elimination interventions. Their inclusion enabled the study to capture perspectives from different levels of the health system and to identify contextual factors influencing the development, implementation, and effectiveness of relevant policies, regulations, and strategies.

Data collection was conducted from April 17, 2023, to May 27, 2023. The principal investigator conducted the qualitative data collection using semi‐structured interview and discussion guides developed in accordance with the study objectives. Key informant interviews, in‐depth interviews, and focus group discussions were conducted with relevant stakeholders and participants, while institutional visits and review of relevant policy, strategic, guideline, and regulatory documents were undertaken to complement the interview data.

### 2.2 Study Design

A facility‐ and institution‐based qualitative study was conducted to explore the challenges and opportunities affecting the development, implementation, and effectiveness of Human Immunodeficiency Virus (HIV), Hepatitis B Virus (HBV), and Hepatitis C Virus (HCV) control and elimination policies, regulations, and strategies in Ethiopia. The study employed an interpretive qualitative approach to obtain in‐depth information from policymakers, program managers, researchers, healthcare professionals, laboratory personnel, pharmacists, and other relevant stakeholders involved in policy development, program implementation, regulation, and service delivery. Data were collected through qualitative interviews and focus group discussions, complemented by a review of relevant policies, strategies, guidelines, and other program documents, as well as observations from selected health‐service and pharmaceutical settings.

### 2.3 Study Participant

The study participants were purposively selected from key stakeholders involved in the development, implementation, regulation, monitoring, and delivery of HIV, HBV, and HCV control and elimination programs in Ethiopia. Participants included policymakers and program managers from the Federal Ministry of Health (MoH), regional health bureaus, and zonal and district health offices; researchers and technical experts from the Ethiopian Public Health Institute (EPHI), Amhara Public Health Institute (APHI), and Ethiopian higher education institutions; healthcare professionals, including physicians, nurses, and other clinical practitioners; laboratory technologists from regional blood bank units and health facilities; pharmacists working in government and private institutions; and other relevant stakeholders involved in HIV, HBV, and HCV prevention, diagnosis, treatment, care, and pharmaceutical supply.

In addition, individuals receiving HIV, HBV, or HCV‐related healthcare services were included to provide perspectives on service accessibility, utilization, experiences, and challenges encountered at the patient and community levels. Relevant policy, program, and strategic documents were also reviewed to complement the information obtained from participants.

Participants were selected based on their knowledge, professional experience, or direct involvement in HIV, HBV, and HCV policy development, implementation, regulation, research, or service delivery. Individuals who met the predefined eligibility criteria and voluntarily provided informed consent to participate during the study period were included in the study (Table 1).

**Table 1:** Distribution of participants involved in the assessment of challenges and opportunities affecting HIV, HBV, and HCV control and elimination policies, regulations, and strategies in Ethiopia, 2023 (*n* = 34).

Stakeholder group	Institution/Organization	Position/Role	No. of participants
**Policymakers and program managers**	Federal Ministry of Health (MoH)	Director/Program manager	2
	Regional Health Bureau (RHB)	Director/Program manager	2
	Gondar City Health Office	Office leader/Program focal person	1
	Central Gondar Zone Health Office	Office leader/Program focal person	1
	North Gondar Zone Health Office	Office leader/Program focal person	1
	West Gondar Zone Health Office	Office leader/Program focal person	1
	Gondar Zuria Health Office	Office leader/Program focal person	1
**Subtotal**			**9**
**Research and technical experts**	Ethiopian Public Health Institute (EPHI)	Researcher/Laboratory expert	2
	Amhara Public Health Institute (APHI)	Researcher/Laboratory expert	2
	University of Gondar (UoG)	Researcher/Academic	2
**Subtotal**			**6**
**Healthcare service providers**	University of Gondar Comprehensive Specialized Hospital	Physician/Doctor	3
	University of Gondar Comprehensive Specialized Hospital	Nurse	2
	University of Gondar Comprehensive Specialized Hospital	Health officer	2
	University of Gondar Comprehensive Specialized Hospital	Laboratory professional	2
	University of Gondar Comprehensive Specialized Hospital	Pharmacist	2
	Gondar Zuria Health Center	Nurse	1
	Gondar Zuria Health Center	Laboratory professional	1
**Subtotal**			**13**
**Blood‐bank and laboratory stakeholders**	Regional Blood Bank	Office leader/Manager	1
	Regional Blood Bank	Laboratory/Blood‐bank officer	1
**Subtotal**			**2**
**Pharmaceutical stakeholders**	Government and private drug stores/pharmacies	Pharmacist/Pharmaceutical personnel	4
**Subtotal**			**4**
**Total**			**34**

Abbreviations: APHI= Amhara Public Health Institute, EPHI= Ethiopian Public Health Institute, HBV= Hepatitis B Virus, HCV= Hepatitis C Virus, HIV= Human Immunodeficiency Virus, MoH= Ministry of Health, RHB= Regional Health Bureau, UoG=University of Gondar.

### 2.4 Sampling and Sampling Procedure

Participants were selected using purposive sampling, a non‐probability sampling technique appropriate for qualitative research. The selection was based on participants’ knowledge, professional experience, roles, and direct involvement in the development, implementation, regulation, monitoring, research, or delivery of HIV, HBV, and HCV control and elimination programs in Ethiopia.

A total of 34 participants were purposively selected from relevant stakeholder groups, including policymakers and program managers, researchers and technical experts, healthcare professionals, laboratory and blood‐bank personnel, pharmacists, and other stakeholders involved in HIV, HBV, and HCV control and elimination. Participant selection was guided by the type of organization, professional role, level of involvement in HIV, HBV, and HCV programs, and relevant experience and expertise.

Recruitment continued until data saturation was reached, defined as the point at which additional interviews and discussions no longer generated substantial new information, concepts, or themes relevant to the study objectives. The diversity of participants across national, regional, subnational, research, clinical, laboratory, pharmaceutical, and service‐delivery settings was intended to capture multiple perspectives on the challenges and opportunities affecting the development, implementation, and effectiveness of HIV, HBV, and HCV control and elimination policies, regulations, and strategies in Ethiopia.

### 2.5 Data Collection Strategy

Data were collected in three sequential phases using qualitative methods to explore the challenges and opportunities influencing the development, implementation, and effectiveness of Human Immunodeficiency Virus (HIV), Hepatitis B Virus (HBV), and Hepatitis C Virus (HCV) control and elimination policies, regulations, and strategies in Ethiopia. The qualitative data collection comprised in‐depth interviews (IDIs), key informant interviews (KIIs), and focus group discussions (FGDs), allowing the study to capture individual experiences, stakeholder perspectives, and shared experiences related to HIV, HBV, and HCV prevention, diagnosis, treatment, care, and elimination.

**Phase 1: In‐Depth Interviews (IDIs)**

In‐depth interviews were conducted with individuals living with HIV, including those receiving antiretroviral therapy (ART), to explore their experiences and perceptions regarding HIV, HBV, and HCV prevention, diagnosis, treatment, care, and related health services. The interviews focused on participants’ experiences with healthcare services, access to information and treatment, perceived barriers to service utilization, experiences with healthcare providers, and factors that facilitate or hinder effective implementation of HIV, HBV, and HCV control and elimination interventions.

Participants were also encouraged to describe their perceptions of existing HIV, HBV, and HCV policies, regulations, and strategies and how these policies translate into services at the community and health‐facility levels. Particular attention was given to challenges related to access, affordability, availability of medicines and diagnostic services, stigma and discrimination, continuity of care, and opportunities for improving implementation.

**Phase 2: Key Informant Interviews (KIIs)**

Key informant interviews were conducted with policymakers, program managers, health‐system leaders, healthcare professionals, researchers, laboratory professionals, pharmacists, and other stakeholders involved in the planning, coordination, regulation, implementation, and monitoring of HIV, HBV, and HCV control and elimination programs in Ethiopia.

The KIIs explored participants’ perspectives on the development and implementation of national HIV, HBV, and HCV policies, regulations, and strategies; stakeholder roles and responsibilities; coordination mechanisms; availability of financial, human, pharmaceutical, laboratory, and other resources; and the current status of implementation. Participants were also asked to identify major barriers and enabling factors affecting policy implementation at national, regional, and service‐delivery levels.

The interviews further explored opportunities for strengthening prevention, case identification, testing and diagnosis, linkage to care, treatment, viral suppression, monitoring, surveillance, and integration of HIV, HBV, and HCV services within the Ethiopian health system. Participants were encouraged to provide recommendations for addressing identified policy, regulatory, organizational, and implementation gaps.

**Phase 3: Focus Group Discussions (FGDs)**

Focus group discussions were conducted with healthcare professionals and other relevant stakeholders involved in HIV, HBV, and HCV prevention, diagnosis, treatment, care, and program implementation. The FGDs facilitated discussion of shared experiences, perceptions, and contextual factors influencing the implementation of HIV, HBV, and HCV control and elimination policies, regulations, and strategies.

The discussions explored health‐system challenges, availability of medicines and diagnostic services, human‐resource capacity, provider knowledge and skills, referral and linkage systems, stigma and discrimination, financing, coordination among stakeholders, monitoring and evaluation, and the integration of HIV, HBV, and HCV services. Participants also discussed opportunities to improve implementation and accelerate progress toward national and global control and elimination targets.

### 2.6 Trustworthiness of the Study

To ensure the trustworthiness and methodological rigor of the qualitative study, the principles of credibility, transferability, dependability, and confirmability were considered throughout the research process.

Credibility was enhanced through the use of multiple qualitative data sources, including in‐depth interviews (IDIs), key informant interviews (KIIs), focus group discussions (FGDs), document review, and direct observation of relevant service and pharmacy settings. These complementary sources enabled triangulation of information and provided opportunities to compare perspectives across different participant groups and sources. The semi‐structured interview and discussion guides were developed based on the study objectives, reviewed by subject‐matter experts, and pre‐tested in a non‐study setting to improve their clarity, relevance, and applicability. Experienced qualitative researchers conducted the data collection following standardized procedures, and regular supervision and debriefing were undertaken to enhance consistency and data quality.

Transferability was supported by providing detailed descriptions of the study context, participant groups, data‐collection procedures, and the policy and health‐system environment in which HIV, HBV, and HCV control and elimination policies, regulations, and strategies were implemented. Such contextual descriptions enable readers and researchers to assess the applicability of the findings to similar settings.

Dependability was strengthened through the systematic application of standardized data‐collection procedures, researcher training, close supervision, and documentation of methodological decisions throughout the study. The development, review, and pre‐testing of the interview guides, together with regular debriefing sessions, helped maintain consistency across IDIs, KIIs, and FGDs. The research team also reviewed transcripts and translated materials for completeness and consistency before analysis.

Confirmability was enhanced by maintaining a close connection between the findings and the original participant accounts. Audio recordings were transcribed verbatim, and transcripts were translated into English by bilingual professionals familiar with the study subject matter. A back‐translation procedure was performed on a subset of transcripts to verify translation accuracy and preserve the intended meaning of participants’ responses. Field notes, documentary evidence, and observations were also considered alongside interview and discussion data. The use of multiple sources and perspectives helped minimize reliance on a single interpretation and strengthened the extent to which the findings were grounded in the collected evidence.

Overall, the combination of data‐source triangulation, expert review and pre‐testing of data‐collection tools, researcher training and supervision, systematic transcription and translation procedures, back‐translation, and detailed documentation of the research process strengthened the credibility, transferability, dependability, and confirmability of the findings concerning the challenges and opportunities affecting HIV, HBV, and HCV control and elimination policies, regulations, and strategies in Ethiopia.

### 2.7 Data Analysis

Data from in‐depth interviews (IDIs), key informant interviews (KIIs), and focus group discussions (FGDs) were analyzed using thematic analysis with a deductive and inductive approach. The analysis was guided by the study objectives and key domains related to HIV, HBV, and HCV control and elimination policies, regulations, and strategies in Ethiopia. Interview and focus group recordings were transcribed verbatim and, where necessary, translated into English. The transcripts, interview notes, relevant strategic and policy documents, and field observations were subsequently organized and imported into MAXQDA Analytics Pro 2024 to facilitate systematic data management, coding, categorization, and retrieval.

An initial coding framework was developed based on the study objectives and the major areas of inquiry, including policy and regulatory frameworks, policy development and implementation, stakeholder roles and responsibilities, health‐system factors, financing, human resources, availability of medicines and commodities, laboratory and diagnostic capacity, service accessibility, monitoring and evaluation, barriers, enabling factors, and opportunities for strengthening HIV, HBV, and HCV control and elimination. Both deductive codes derived from these predefined domains and inductive codes emerging directly from participants’ accounts and reviewed documents were used during the analysis.

The analysis was conducted iteratively. Transcripts and other qualitative materials were carefully reviewed to identify meaningful statements and recurring patterns. Relevant segments of text were coded and subsequently organized into categories and sub‐themes. Related codes were then grouped into broader themes representing major challenges, enabling factors, policy and implementation gaps, and opportunities for strengthening HIV, HBV, and HCV control and elimination in Ethiopia. Themes were continuously reviewed, refined, and compared against the original data to ensure that they accurately reflected participants’ perspectives and the documentary evidence.

Information obtained from IDIs, KIIs, FGDs, policy and strategic documents, and field observations was analyzed and compared to identify areas of convergence, divergence, and gaps. Triangulation across data sources and data‐collection methods was used to strengthen the credibility and comprehensiveness of the findings. Particular attention was given to differences and similarities in perspectives among service users, policymakers, program managers, healthcare professionals, researchers, laboratory personnel, pharmacists, and other relevant stakeholders.

Throughout the analysis, the research team engaged in regular analytical discussions to critically examine emerging interpretations, assumptions, and contextual explanations. The researchers independently reviewed relevant qualitative materials and subsequently discussed the findings to refine the coding framework, resolve differences in interpretation, and reach agreement on the scope and meaning of the emerging themes. Rather than relying solely on numerical measures of coding agreement, the team used iterative discussion and reflexive interpretation to strengthen analytical depth and consistency.

Finally, the identified themes and sub‐themes were synthesized to explain the challenges and opportunities affecting the development, implementation, and effectiveness of HIV, HBV, and HCV control and elimination policies, regulations, and strategies in Ethiopia. Findings from participant interviews and discussions were integrated with evidence from policy and strategic documents and field observations to provide a comprehensive understanding of policy gaps, implementation barriers, enabling factors, and opportunities for strengthening the national HIV, HBV, and HCV response.

## 3 Results and Discussion

### 3.1 Participant Characteristics

A total of 34 participants were included in the qualitative study. The participants represented diverse stakeholder groups involved in the development, implementation, regulation, research, and delivery of HIV, HBV, and HCV control and elimination interventions in Ethiopia. The mean duration of participants’ professional work experience was 16.82 years (SD = 0.79), indicating substantial professional experience among the informants.

The majority of participants were male (30, 88%), while 4 (12%) were female. Regarding educational attainment, 6 (18%) participants had a PhD or postdoctoral‐level qualification, 19 (56%) had a master's‐level qualification (MPH/MSc), and 9 (26%) had a bachelor's‐level qualification (BSc).

In terms of professional background, 15 (44%) were policymakers or program managers, 4 (12%) were researchers, 2 (6%) were physicians, 1 (3%) was a health officer, 4 (12%) were nurses, 3 (9%) were laboratory technologists, and 5 (14%) were pharmacists. Regarding organizational positions, 2 (6%) participants were directors, 6 (18%) were office leaders, 7 (21%) were coordinators, and 19 (56%) were officers.

With respect to professional experience, 8 (24%) participants had 5–15 years of experience, 16 (47%) had 16–25 years, and 10 (29%) had more than 26 years of professional experience. Overall, the participants had diverse professional backgrounds and substantial experience, enabling the study to capture multiple perspectives on the challenges and opportunities affecting the development, implementation, and effectiveness of HIV, HBV, and HCV control and elimination policies, regulations, and strategies in Ethiopia (Table 2).

**Table 2:** Characteristics of participants involved in the assessment of HIV, HBV, and HCV control and elimination policies, regulations, and strategies in Ethiopia, 2023 (*n* = 34).

Characteristics	Frequency (*n*)	Percent (%)
**Sex**		
Male	30	88
Female	4	12
**Educational level**		
PhD/Postdoctoral level	6	18
MPH/MSc level	19	56
BSc level	9	26
**Professional background**		
Policymaker/Program manager	15	44
Researcher	4	12
Physician	2	6
Health officer	1	3
Nurse	4	12
Laboratory technologist	3	9
Pharmacist	5	14
**Position**		
Director	2	6
Office leader	6	18
Coordinator	7	21
Officer	19	56
**Years of professional experience**		
5–15 years	8	24
16–25 years	16	47
> 26 years	10	29
**Total**	**34**	**100**

Abbreviations: BSc= Bachelor of Science, HBV= Hepatitis B Virus, HCV= Hepatitis C Virus, HIV= Human Immunodeficiency Virus, MPH= Master of Public Health, MSc, Master of Science.

### 3.2 Thematic Analysis

The qualitative data were analyzed thematically to identify recurring concepts and patterns related to the development, implementation, and effectiveness of HIV, HBV, and HCV control and elimination policies, regulations, and strategies in Ethiopia. Six major thematic areas emerged from the analysis [1]: features of existing policies, regulations, and strategies [2]; initiatives for HIV, HBV, and HCV control and elimination [3]; roles and responsibilities of stakeholders [4]; current implementation status [5]; opportunities for strengthening the response; and [6] challenges affecting implementation.

Among the 34 participants, 12 (35.3%) reported or demonstrated adequate awareness of the features of existing HIV, HBV, and HCV control and elimination policies, regulations, and strategies, while 22 (64.7%) did not identify or demonstrate clear awareness of these features. Regarding initiatives for disease control and elimination, 15 (44.1%) participants identified relevant initiatives, whereas 19 (55.9%) did not.

Most participants, 32 (94.1%), identified clear roles and responsibilities of stakeholders in the HIV, HBV, and HCV control and elimination response, while only 2 (5.9%) did not. In contrast, only 8 (23.5%) participants considered the implementation status of the policies, regulations, and strategies to be satisfactory or adequately established, while 26 (76.5%) identified gaps in their implementation status.

Regarding opportunities for strengthening the response, 5 (14.7%) participants identified specific opportunities, whereas 29 (85.3%) did not identify sufficient opportunities within the areas assessed. In relation to challenges affecting the response, 32 (94.1%) participants identified important challenges influencing the implementation of HIV, HBV, and HCV control and elimination policies, regulations, and strategies, while only 2 (5.9%) did not report major challenges (Figure 1).

**Figure 1:**
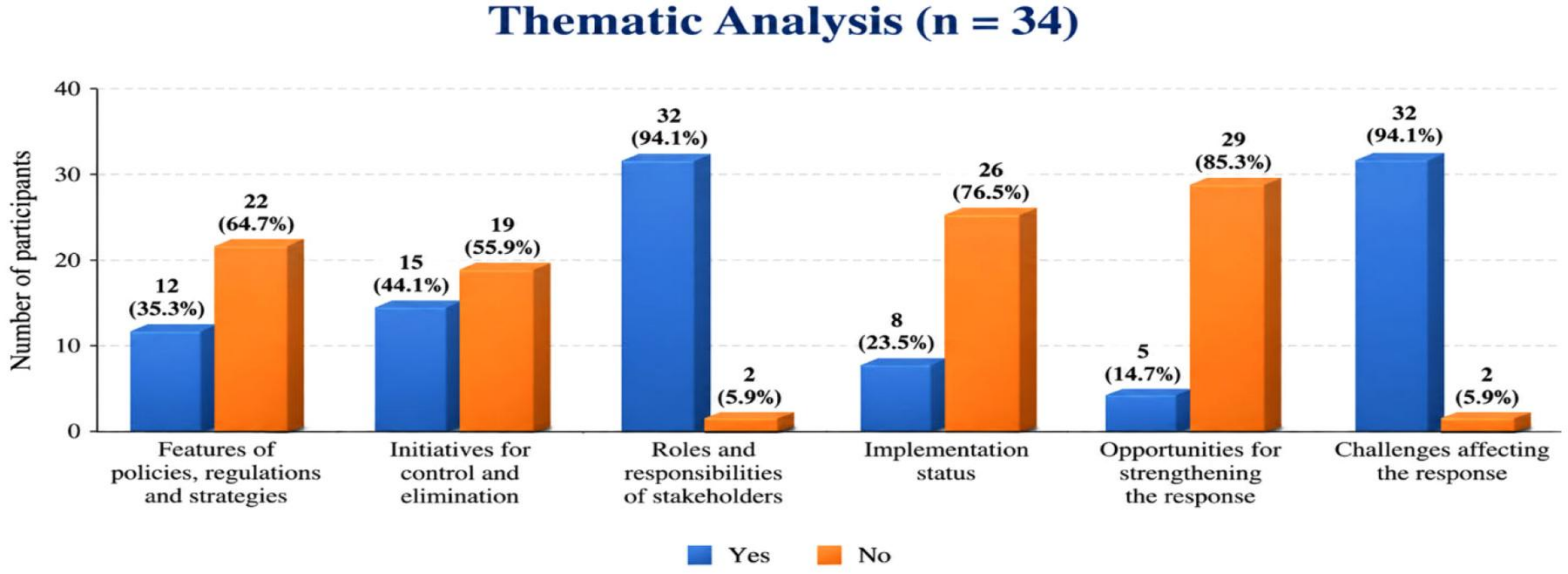
Thematic analysis of participants’ perspectives on the challenges and opportunities affecting HIV, HBV, and HCV control and elimination policies, regulations, and strategies in Ethiopia, 2023 (*n* = 34). Blue bars (Yes) represent participants who identified or affirmed the respective thematic area, while orange bars (No) represent those who did not. Values above the bars indicate the number and percentage of participants responding to each thematic area. Percentages were calculated using the total sample of 34 participants. HBV = Hepatitis B Virus, HCV = Hepatitis C Virus,HIV = Human Immunodeficiency Virus.

Among the 34 participants, 12 (35.3%) identified or demonstrated awareness of the features and existence of relevant policies, regulations, and strategies, whereas 22 (64.7%) did not. Regarding initiatives for HIV, HBV, and HCV control and elimination, 15 (44.1%) participants identified relevant initiatives, while 19 (55.9%) did not.

The majority of participants, 32 (94.1%), identified clear roles and responsibilities of stakeholders in the HIV, HBV, and HCV response, whereas 2 (5.9%) did not. Regarding the current implementation status of policies, regulations, and strategies, only 8 (23.5%) participants reported or identified evidence of implementation, while 26 (76.5%) identified gaps or limitations in the current status.

Only 5 (14.7%) participants identified specific opportunities for strengthening the HIV, HBV, and HCV response, whereas 29 (85.3%) did not identify sufficient opportunities within the areas explored. In contrast, 32 (94.1%) participants identified challenges affecting the implementation and effectiveness of HIV, HBV, and HCV control and elimination policies, regulations, and strategies, while 2 (5.9%) did not report major challenges (Table 3).

**Table 3:** Participants’ responses regarding HIV, HBV, and HCV control and elimination policies, regulations, and strategies in Ethiopia, 2023 (*n* = 34).

Thematic area	Response	Frequency (*n*)	Percent (%)
**Features and existence of policies, regulations, and strategies**	Yes	12	35.3
	No	22	64.7
**Initiatives for HIV, HBV, and HCV control and elimination**	Yes	15	44.1
	No	19	55.9
**Roles and responsibilities of stakeholders**	Yes	32	94.1
	No	2	5.9
**Current implementation status**	Yes	8	23.5
	No	26	76.5
**Opportunities for strengthening the response**	Yes	5	14.7
	No	29	85.3
**Challenges affecting the response**	Yes	32	94.1
	No	2	5.9

*Note:* “Yes” indicates that participants identified, reported, or acknowledged the respective thematic area during the qualitative assessment, while “No” indicates that the respective issue was not identified or reported by the participant. Percentages were calculated using the total number of participants (*n* = 34).

Abbreviations: HBV= Hepatitis B Virus, HCV= Hepatitis C Virus, HIV, Human Immunodeficiency Virus.

#### 3.2.1 Participants’ Perspectives on the Existence and Features of Policies, Regulations, and Strategies

The findings showed variation in participants’ awareness of the existence and key features of HIV, HBV, and HCV control and elimination policies, regulations, and strategies in Ethiopia. Among the 34 participants, 12 (35.3%) identified or described the existence and key features of relevant policies, regulations, and strategies, whereas 22 (64.7%) did not clearly identify or describe these policies, regulations, or strategies.

Participants who demonstrated awareness described the policies and strategies in relation to their roles in policy development, program coordination, implementation, monitoring, and evaluation. In contrast, other participants were unable to clearly identify the relevant policy and strategy documents or describe their key features. The level of awareness varied across participants according to their institutional roles, involvement in HIV, HBV, and HCV programs, and exposure to relevant policy and program activities.

One participant expressed uncertainty regarding the existence and practical implementation of hepatitis control and elimination policies and strategies, stating: “*Are you saying there is a hepatitis control and elimination policy and strategy program? Please do not lead me to laugh, please do not lead me to laugh, please do not lead me to laugh. Everything in our country looks like it is only for show. You can check with the Regional Health Bureau and the Ministry of Health; they might say something for show*.” (Participant 21)

The participant's account illustrates the uncertainty expressed by some stakeholders regarding the visibility and practical implementation of hepatitis‐related policies and strategies. Overall, the qualitative findings indicated that while some participants were familiar with the existence and features of HIV, HBV, and HCV policies, regulations, and strategies, a substantial proportion could not clearly identify or describe them.

#### 3.2.2 Initiation and Stakeholder Involvement in HIV, HBV, and HCV Policy, Regulation, and Strategy Development

Participants described the involvement of multiple stakeholders in the development of HIV, HBV, and HCV control and elimination policies, regulations, and strategies, including policymakers, healthcare service providers, researchers, professional associations, non‐governmental organizations (NGOs), development partners, WHO, and other relevant government institutions.

Among the 34 participants, 15 (44.1%) identified the involvement of stakeholders such as WHO, NGOs, professional associations, researchers, and healthcare service providers in the development of HIV, HBV, and HCV control and elimination policies, regulations, and strategies. In contrast, 19 (55.9%) participants did not identify or report adequate opportunities for stakeholder participation in the policy and strategy development process.

Participants reported differences in stakeholder involvement across institutional and administrative levels. Some participants described engagement in policy and strategy development, whereas others reported limited involvement or lack of opportunities to contribute to the development process, particularly among stakeholders outside the central level.

One participant expressed concern regarding the visibility and awareness of hepatitis‐related policies and programs, stating: “*Are you saying there is a hepatitis control and elimination policy and strategy program? Please do not lead me to laugh, please do not lead me to laugh, please do not lead me to laugh. Everything in our country looks like it is only for show. You can check with the Regional Health Bureau and the Ministry of Health; they might say something for show*.” (**Participant 21**)

Participants also reported limited engagement in policy‐development activities, including opportunities to provide research findings and other evidence during the preparation of policies, regulations, and strategies. Some participants mentioned that they had not received invitations or opportunities to contribute their experiences, evidence, or technical perspectives during the development process.

#### 3.2.3 Role of Stakeholders in HIV, HBV, and HCV Policy, Regulation, and Strategy Development and Implementation

The majority of participants, 32 (94.1%), reported a clear understanding of their roles and responsibilities in the development and implementation of HIV, HBV, and HCV policies, regulations, and strategies. Participants described stakeholder responsibilities as including policy and strategy formulation, involvement of relevant organizations, awareness creation, resource mobilization, capacity building, training, and support for implementation.

One policymaker stated: “*Our responsibility is formulation of hepatitis elimination policies, regulations, and strategies with the involvement of different stakeholders, such as NGOs, WHO, and different professional associations; creating awareness; facilitating mobilization; arranging different trainings; and searching for different funding options for the implementation of policies, regulations, and strategies*.” (**Participant 02**).

Participants further described the involvement of government institutions, nongovernmental organizations, WHO, professional associations, researchers, healthcare professionals, and other relevant stakeholders as important components of policy and strategy development and implementation. The findings indicated that stakeholder roles were perceived to extend from policy formulation to awareness creation, training, coordination, resource mobilization, and implementation of planned interventions.

#### 3.2.4 Current Implementation Status of HIV, HBV, and HCV Policies, Regulations, and Strategies

Participants reported varying perceptions regarding the current implementation status of HIV, HBV, and HCV control and elimination policies, regulations, and strategies in Ethiopia. Among the 34 participants, 8 (23.5%) reported or described the current implementation status and progress of the policies, regulations, and strategies. In contrast, 26 (76.5%) participants reported that there was no clear implementation status or identifiable progress regarding the relevant policies, regulations, and strategies.

Participants who reported an implementation status described the existence of ongoing activities related to the control and elimination of HIV, HBV, and HCV. However, other participants reported uncertainty regarding the existence, implementation, or progress of the relevant policies, regulations, and strategies.

One participant expressed uncertainty regarding the existence and implementation of hepatitis control and elimination policies, regulations, and strategies, stating: “*Why are you asking these questions since there is no hepatitis control and elimination policy, regulation, and strategies in the country? It is better to ask policymakers and politicians because the document might be there in their office*.” (Participant 23). Overall, the findings showed that 8 (23.5%) participants reported a current implementation status or progress, whereas 26 (76.5%) participants did not identify a clear implementation status or progress of HIV, HBV, and HCV control and elimination policies, regulations, and strategies.

#### 3.2.5 Opportunities for HIV, HBV, and HCV Policy, Regulation, and Strategy Implementation

Participants reported limited opportunities related to the implementation of HIV, HBV, and HCV control and elimination policies, regulations, and strategies in Ethiopia. Among the 34 participants, 29 (85.3%) reported that there were limited or no clear opportunities for the implementation and strengthening of hepatitis elimination policies, regulations, and strategies. In contrast, 5 (14.7%) participants identified opportunities related to HIV, HBV, and HCV control and elimination.

Participants described the opportunities in relation to the implementation and strengthening of existing policies, regulations, and strategies. However, the majority reported that opportunities were limited in the context of the challenges affecting HIV, HBV, and HCV control and elimination activities.

One key informant stated: “*How can we have any opportunity for hepatitis elimination policies, regulations, and strategies when there are so many challenges*?” (**Participant 16**). Overall, the findings showed that most participants, 29 (85.3%), did not identify clear opportunities for HIV, HBV, and HCV control and elimination policies, regulations, and strategies, while 5 (14.7%) participants reported the presence of opportunities.

#### 3.2.6 Challenges Affecting HIV, HBV, and HCV Policies, Regulations, and Strategies

Participants reported several challenges affecting the implementation of HIV, HBV, and HCV control and elimination policies, regulations, and strategies in Ethiopia. Among the 34 participants, 32 (94.1%) identified or described challenges affecting the implementation of policies, regulations, and strategies, whereas 2 (5.9%) participants did not clearly report specific challenges.

Participants described challenges related to the implementation of hepatitis control and elimination activities, including the effects of the COVID‐19 pandemic on hepatitis B and C control and elimination activities. One healthcare service provider stated: “*The impact of COVID‐19 highly affected hepatitis B and C virus control and elimination in Ethiopia*.”

Another healthcare service provider expressed concerns regarding the implementation and reporting of health policies and strategies, stating: “*Every Ethiopian policy and strategy is being implemented for show and reporting to WHO rather than in real practice*.” (**Participant 18**)

Overall, the findings showed that the majority of participants, 32 (94.1%), identified challenges affecting HIV, HBV, and HCV control and elimination policies, regulations, and strategies. The reported challenges included disruptions associated with the COVID‐19 pandemic and concerns regarding the implementation of policies and strategies in practice.

## 4 Discussion

This study explored stakeholder perspectives on the development, implementation, opportunities, and challenges related to HIV, HBV, and HCV control and elimination policies, regulations, and strategies in Ethiopia. The findings demonstrate an important discrepancy between recognition of stakeholder responsibilities and the practical visibility and implementation of policies. Although 94.1% of participants demonstrated an understanding of stakeholder roles, only 35.3% clearly identified the existence and key features of relevant policies, 44.1% reported stakeholder involvement in policy and strategy development, and only 23.5% reported a clear current implementation status. Furthermore, 85.3% perceived limited opportunities for strengthening hepatitis elimination activities, while 94.1% identified challenges affecting implementation. Taken together, these findings suggest that the principal issue is not simply the absence of policy documents, but the extent to which policies are communicated, translated into operational activities, monitored, financed, and coordinated across different levels of the health system.

### 4.1 Awareness of HIV, HBV, and HCV Policies, Regulations, and Strategies

Only 35.3% of participants in this study clearly identified or described the existence and key features of HIV, HBV, and HCV control and elimination policies, regulations, and strategies, whereas 64.7% did not clearly identify or describe them. This finding is consistent with earlier Ethiopian evidence showing that, although Ethiopia had begun developing hepatitis strategic plans and guidelines, the national response was characterized by limited coordination, inadequate dissemination of guidance, weak surveillance, and limited access to hepatitis services. In their qualitative assessment of Ethiopia's hepatitis response, Abera et al. reported that the country was developing strategic plans and guidelines but lacked sufficient partnerships and resource mobilization, while treatment and screening guidelines were not adequately available or implemented [5].

The finding is also consistent with more recent evidence showing that knowledge and awareness do not necessarily translate into appropriate hepatitis‐related practices in Ethiopia. For example, a systematic review and meta‐analysis of 26 Ethiopian studies reported that only 47.45% of participants had a good level of knowledge concerning HBV prevention, while good preventive practices were reported in only 36.22% (2026). Although that evidence concerns individual‐level knowledge rather than awareness of national policies, it supports the broader observation that information and communication gaps remain relevant to hepatitis prevention and control in Ethiopia.

Evidence from other settings similarly demonstrates that policy formulation does not automatically ensure effective policy awareness or implementation. A global assessment of 55 national viral hepatitis strategic action plans found substantial gaps in alignment with WHO recommendations: 35% of plans did not include HBV and HCV elimination goals, and only 38% included a national budget or financing plan for hepatitis activities [7]. More recent country profiles also show continuing weaknesses in national monitoring and service coverage despite the existence of formal policies. Among 33 countries assessed for HCV elimination, only 55% had systems tracking the number of people tested and treated, while only 30% had achieved the WHO 2025 diagnosis‐coverage target [7, 8].

The Ethiopian finding should therefore be interpreted as an implementation‐system issue rather than simply an individual knowledge deficit. The WHO Global Health Sector Strategy 2022–2030 emphasizes that countries need effective strategic information systems capable of generating and analyzing data from service‐delivery and administrative levels to monitor progress toward elimination targets [1]. The low awareness observed in the present study may therefore reflect limited dissemination and operational communication of policy documents between national, regional, and service‐delivery levels.

### 4.2 Stakeholder Involvement in Policy and Strategy Development

Only 44.1% of participants reported involvement of relevant stakeholders in the development of HIV, HBV, and HCV policies, regulations, and strategies, while 55.9% did not report adequate opportunities for participation. This finding is important because WHO's 2022–2030 Global Health Sector Strategies explicitly emphasize leadership, partnerships, multisectoral action, community engagement, and collaboration with stakeholders as central components of the response to HIV and viral hepatitis [1]. The WHO strategy also describes its own development process as broadly consultative, involving Member States, experts, communities, partners, and other stakeholders.

The relatively limited stakeholder involvement identified in this study is consistent with the earlier Ethiopian qualitative assessment by Abera et al., which identified inadequate partnerships and limited resource mobilization as important weaknesses in the country's hepatitis response [5]. It also corresponds with evidence from sub‐Saharan Africa showing that operationalizing hepatitis elimination requires coordinated involvement of governments, healthcare providers, technical organizations, professional groups, and development partners. African countries have faced difficulties translating HCV elimination objectives into operational programs because of diagnostic costs, medicine access, logistics, and limited program infrastructure [9].

There are also examples demonstrating the potential value of coordinated national and continental approaches. The establishment of the Africa CDC viral hepatitis prevention and control program represents a continental mechanism intended to support countries in developing and strengthening hepatitis elimination program [10]. Similarly, Rwanda and Egypt are among countries that have demonstrated progress in operationalizing hepatitis elimination approaches, although their experiences occur within different health‐system and financing contexts and should not be directly equated with Ethiopia.

The finding from this study is therefore relevant to WHO's emphasis on a whole‐of‐government and whole‐of‐society approach. The global strategy recognizes that effective implementation requires political and community leadership and partnerships extending beyond the health sector [1]. Limited participation of stakeholders outside central institutions may reduce opportunities for incorporating service‐delivery experience, research evidence, professional expertise, and community perspectives into policy development.

### 4.3 Understanding of Stakeholder Roles

A particularly notable finding was that 94.1% of participants demonstrated an understanding of their roles and responsibilities in HIV, HBV, and HCV policy development and implementation. This relatively high recognition contrasts with the lower levels of policy awareness and implementation‐status awareness observed in the same study. Thus, participants appeared to understand what their responsibilities were, even when they had less certainty about which policies were currently being implemented and how much progress had been achieved.

This distinction is important in interpreting the findings. The Ethiopian evidence indicates that healthcare professionals can possess substantial hepatitis‐related knowledge while important gaps remain in translating knowledge into practice. For example, a study among healthcare professionals at the University of Gondar Hospital found that 73.1% had good knowledge of HBV transmission, progression, and vaccination, but knowledge varied substantially by professional group [11]. Similarly, the more recent Ethiopian study of healthcare workers found that only 32.5% demonstrated adequate knowledge concerning HBV post‐exposure prophylaxis despite generally positive attitudes.

The present finding therefore suggests that awareness of professional responsibility alone may not be sufficient to ensure implementation. WHO's global strategy places stakeholder leadership and partnerships alongside health‐system strengthening, strategic information, service integration, and sustainable financing [1]. In other words, individuals may recognize their responsibilities while remaining constrained by institutional systems, financing, commodities, laboratory capacity, monitoring systems, or unclear implementation mechanisms.

This interpretation is also consistent with evidence from Ethiopia showing that hepatitis B vaccination among healthcare professionals has been constrained by practical barriers. A national systematic review found pooled full‐dose HBV vaccination coverage among Ethiopian healthcare workers of only 20.04%, despite the occupational importance of hepatitis prevention [12]. This illustrates the distinction between recognizing responsibility and having the institutional conditions necessary to perform that responsibility.

### 4.4 Current Implementation Status of Policies, Regulations, and Strategies

Only 23.5% of participants were able to identify or describe a clear current implementation status, while 76.5% reported that the implementation status or progress of HIV, HBV, and HCV policies and strategies was unclear. This finding is particularly important because implementation monitoring is a central component of the WHO elimination framework.

The WHO Global Health Sector Strategies 2022–2030 require countries to monitor progress toward defined elimination targets and strengthen strategic information systems. However, the WHO 2024 progress report found that although access to HIV, viral hepatitis, and STI services had improved in some areas, many indicators remained off track for the 2025 and 2030 targets. The latest WHO 2026 mid‐term assessment similarly concludes that global progress is mixed and that the world is off track to meet the 2030 targets, with viral hepatitis deaths increasing rather than declining [3].

The present finding is also compatible with international evidence concerning weaknesses in national monitoring systems. Among 33 national HCV elimination profiles, only 33% had systems to monitor hepatitis C‐related mortality, 48% had systems to monitor incidence, and 55% had systems tracking the number of people tested and treated [8]. These findings indicate that having a national policy or action plan does not necessarily mean that implementation progress is systematically measured.

Evidence from Ethiopia provides an additional explanation for why implementation status may be difficult to determine. The earlier Ethiopian policy assessment identified inadequate surveillance and limited diagnostic infrastructure as important weaknesses in the national hepatitis response [5]. More recent modeling work estimated approximately 7.6 million HBV infections and 690,000 HCV infections in Ethiopia in 2023 and concluded that substantial additional investment would be required to expand diagnosis and treatment to achieve elimination targets. These findings reinforce the importance of reliable program data for determining whether policy commitments are translating into measurable service coverage and health outcomes.

### 4.5 Opportunities for HIV, HBV, and HCV Elimination

In this study, 85.3% of participants reported limited or unclear opportunities for HIV, HBV, and HCV elimination, whereas only 14.7% identified clear opportunities. This perception contrasts with the substantial opportunities identified in international policy and scientific literature.

At the global level, WHO identifies several evidence‐based opportunities for accelerating viral hepatitis elimination, including vaccination, prevention of mother‐to‐child transmission, expanded diagnosis and treatment, integration with primary healthcare, improved strategic information, and stronger partnerships [1, 2]. WHO's 2026 progress report further identifies integrated services, stronger community leadership, improved use of integrated health data, protection of essential services during disruptions, and strategically targeted investments as important mechanisms for accelerating progress.

The contrast between these established opportunities and the limited opportunities perceived by participants may reflect a gap between policy‐level opportunities and implementation‐level opportunities. In Ethiopia, for example, recent modeling suggests that expanding universal hepatitis B birth‐dose vaccination and screening of pregnant women could substantially reduce HBV transmission, while expanding annual HCV screening and treatment could move the country toward elimination. Similarly, a proposed Ethiopian HCV micro‐elimination approach identified task shifting, affordable testing, access to direct‐acting antivirals, blood safety, multisectoral partnerships, and regulatory support as potential enabling mechanisms [6].

African evidence also demonstrates that opportunities can be converted into measurable program gains when appropriate systems are established. Rwanda, Egypt, and several other African countries have been cited as examples of countries making progress toward HCV elimination, although their approaches have occurred under different financing, governance, and health‐system conditions [9]. Thus, the 85.3% perception of limited opportunities in the present study should not be interpreted as evidence that opportunities objectively do not exist. Rather, it suggests that many opportunities identified at global or policy levels may not yet be sufficiently visible or operationalized to stakeholders working within the Ethiopian health system.

### 4.6 Challenges Affecting Policy and Strategy Implementation

Almost all participants, 94.1%, identified challenges affecting HIV, HBV, and HCV control and elimination policies, regulations, and strategies. Participants particularly described the effects of COVID‐19 and concerns about the gap between formal policy commitments and practical implementation.

The reported effect of COVID‐19 is strongly supported by international evidence. A multicounty assessment of viral hepatitis services in Burkina Faso, Tanzania, and The Gambia documented reductions of more than 70% in outpatient hepatitis service use when April 2020 was compared with January 2020 [13]. A global analysis similarly estimated substantial disruption to HCV elimination activities during the pandemic [14]. An international EASL survey found reductions in HBV and HCV consultations, referrals, testing, and treatment during the pandemic, demonstrating that the disruption extended across multiple stages of the hepatitis care cascade (2022). WHO also reported that hepatitis B and C services were among the essential health services disrupted during the COVID‐19 pandemic [1].

These international findings are particularly relevant to the present study because participants specifically described COVID‐19 as affecting hepatitis B and C control and elimination in Ethiopia. The pandemic therefore represents not simply an isolated external event but a disruption that exposed the fragility of hepatitis program, particularly where services are dependent on limited personnel, laboratory capacity, financing, and disease‐specific infrastructure.

Beyond COVID‐19, the challenges identified by participants are consistent with broader evidence from sub‐Saharan Africa. African HCV elimination program face difficulties related to expensive diagnostic platforms, medicine access, program logistics, and the availability of direct‐acting antivirals within national systems [9]. The broader literature similarly identifies financial constraints, limited laboratory infrastructure, centralized models of care, fragmented disease program, and health‐system weaknesses as persistent barriers to elimination in low‐ and middle‐income countries.

The Ethiopian context demonstrates similar structural constraints. Abera et al. previously reported inadequate diagnostic facilities, weak surveillance, limited treatment availability, insufficient partnerships, and inadequate resource mobilization in Ethiopia's hepatitis response [5]. The proposed Ethiopian HCV micro‐elimination framework subsequently identified shortages of qualified personnel and advanced laboratory capacity, healthcare‐associated transmission, blood‐safety concerns, access to affordable diagnostics and DAAs, and the need for multisectoral partnerships as important considerations [6]. More recent economic modeling further indicates that Ethiopia will require substantial investment to expand HBV diagnosis, prevention, and treatment and HCV screening and treatment at the scale required for elimination (2025).

The participant statement that policies and strategies may sometimes be implemented primarily for reporting rather than through observable practice should be interpreted as a stakeholder perception, rather than as an independently verified characterization of the national health system. Nevertheless, it identifies an important implementation concern: the potential gap between policy formulation, reporting, and measurable service delivery. The discrepancy between high recognition of stakeholder roles (94.1%) and low awareness of current implementation status (23.5%) in this study further supports the importance of examining not only whether policies exist, but also whether implementation processes, financing, accountability, monitoring, and service‐delivery mechanisms are functioning.

### 4.7 Overall Interpretation in Relation to the WHO Elimination Framework

Taken together, the findings indicate that Ethiopia has important foundations for HIV, HBV, and HCV elimination, including stakeholder recognition of responsibilities and the existence of national strategic planning. The Ethiopian Ministry of Health currently identifies a separate National Viral Hepatitis Strategic Plan for 2021–2025, with the stated goal of eliminating viral hepatitis as a major public health problem in Ethiopia by 2030. However, the qualitative findings indicate that policy existence has not necessarily translated into broad stakeholder awareness, inclusive participation, clearly visible implementation progress, or perceived opportunities for program strengthening.

This distinction is consistent with the broader international experience. The WHO 2024 global report emphasizes that substantial gaps remain in diagnosis, treatment, prevention, and access to health products, particularly in low‐ and middle‐income countries. The 2026 WHO mid‐term review provides an even stronger indication that policy commitments alone are insufficient: global progress remains off track and viral hepatitis deaths are increasing. Consequently, the Ethiopian findings should be understood within a wider global implementation gap rather than as an isolated national problem.

The evidence suggests that successful implementation requires alignment between policy development and operational systems. WHO's 2022–2030 strategy emphasizes five strategic directions, including service integration, financing, innovation, partnerships, and strategic information, while specifically promoting a whole‐of‐government and whole‐of‐society approach. The findings of this study correspond closely with these domains: limited policy awareness relates to communication and strategic information; limited stakeholder involvement relates to partnerships and leadership; unclear implementation status relates to monitoring and accountability; limited perceived opportunities relate to financing and operational capacity; and the reported challenges reflect health‐system and service‐disruption constraints.

## 5 Strengths and Limitations of the Study

The strengths of the study were the availability of various guidelines, policies, strategies, standards, and study documents, which provided a solid foundation for the study. Additionally, the cooperation of some key informants was invaluable in gathering important insights.

However, there were several limitations that impacted the study. Many key informants were not fully cooperative, often unwilling to provide complete information or participate in recordings. Time constraints and limited financial resources further hindered the study process, making it more difficult to gather comprehensive data.

## 6 Conclusion

Most stakeholders have no information about the availability of hepatitis control and elimination policy, regulations, and strategies, but they effectively understand their role during preparations and implementations of hepatitis control and elimination policy, regulations, and strategies. The policy, regulations, and strategies were not prepared with full involvement of concerned bodies as a result, there is no enough implementations progress reports from the respondents. The existence of hepatitis different guidelines, policies, regulations, strategies, documents, and hepatitis elimination program incorporation to sustainable development goals and vaccine availability for healthcare providers and newborn babies are a good opportunity. Funding problem, lack of training, community culture and belief, COVID‐19, commitments of different stakeholders including government, shortage of medications, lack of awareness and mobilizations, and weak practices of monitoring and evaluations are the most common challenges. Although the COVID‐19 pandemic required immediate public health measures, its effects on hepatitis control initiatives underscore the necessity of comprehensive approaches that tackle both COVID‐19 and persistent viral hepatitis issues. Based on the finding we recommend to revised the existing policy, regulations and strategies with the full invitations of concerned bodies, collaborate with many donor organizations, accessing screening, testing and treatment of the patients, practical performing of monitoring and evaluations, increase the awareness and mobilizations of for patients, health care providers and other concerned bodies are important activities to control and eliminate hepatitis.

## Author Contributions

**Mequanente Dagnaw:** visualization, writing – review and editing, conceptualization, investigation, project administration, resources. **Bisrat Misganaw Geremew:** conceptualization, funding acquisition, writing – review and editing, data curation. **Lemma Derseh Gezie:** data curation, investigation, validation.

## Funding

The authors have nothing to report.

## Ethics Statement

Ethical clearance for this study was obtained from the Institutional Review Board of the IRERC (Ref: CMHSSH‐UoG‐IRERC/108/11/2025; dated 12 November 2025). Written informed consent was obtained from all participants prior to their inclusion in the study. Participant confidentiality was strictly maintained through the use of coded identifiers, and all data were securely stored with access limited to authorized personnel only. Interviews were conducted in a private setting to ensure privacy and comfort. Participation in the study was entirely voluntary, and participants were informed of their right to withdraw at any time without any consequences to their care or services. The study was conducted in accordance with the principles of the Declaration of Helsinki, as well as applicable national and institutional ethical guidelines.

## Consent

Written informed consent was obtained from all participants. They were informed about the study purpose, procedures, risks, benefits, confidentiality, and their right to participate or withdraw without affecting their care. For those unable to read or write, the consent form was explained in the local language and documented by thumbprint with an impartial witness. Participants were allowed to ask questions before consent was confirmed.

## Conflicts of Interest

The authors declare no conflicts of interest.

## Transparency Statement

Mequanente Dagnaw affirms that this manuscript is an honest, accurate, and transparent account of the study being reported; that no important aspects of the study have been omitted; and that any discrepancies from the study as planned have been explained.

## Data Availability

The datasets generated during and/or analyzed during the current study are available from the corresponding author on reasonable request.
